# Micropropagation, Micromorphological Studies, and* In Vitro* Flowering in* Rungia pectinata* L.

**DOI:** 10.1155/2016/5813851

**Published:** 2016-05-08

**Authors:** Mahipal S. Shekhawat, M. Manokari, C. P. Ravindran

**Affiliations:** Biotechnology Laboratory, Department of Plant Science, MGGAC, Mahe, Pondicherry 673 311, India

## Abstract

A tissue culture protocol was developed for an important medicinal plant* Rungia pectinata* L. in the present study. Nodal shoots were used as explants and surface-sterilized with 0.1% HgCl_2_ solution. Murashige and Skoog (MS) medium was used to establish the cultures of* R. pectinata*. The bud break was reported on MS medium supplemented with 1.0 mg L^−1^ 6-benzylaminopurine (BAP). About 98% response was observed with this media combination and maximum 3.2 shoots per explant with 4.3 cm length were recorded. The shoots were further multiplied using MS medium augmented with 0.5 mg L^−1^ each of BAP and kinetin (Kin) + 0.1 mg L^−1^ indole-3 acetic acid (IAA). Maximum 13.2 shoots per explant with 5.2 cm length were observed. All the shoots were rooted (4.9 roots per shoot with 3.5 cm length) on half strength MS medium fortified with 2.0 mg L^−1^ indole-3 butyric acid (IBA).* In vitro* flowering was induced from the shoots on half strength MS medium supplemented with same concentrations and combinations of growth regulators used for shoot multiplication under 12/12 hr light/dark photoperiod. The plantlets were hardened in the greenhouse for two months and finally transferred to the field. The foliar micromorphological studies revealed the developmental changes in stomata, vein density, and trichomes during the culture of shoots under* in vitro* conditions.

## 1. Introduction

*Rungia pectinata* L. is an ethnomedicinal plant which belongs to the family Acanthaceae.* R. parviflora* var.* pectinata*,* R. parviflora* var.* muralis*, and* Justicia pectinata* are the synonyms of this plant. It is an annual erect herb with profuse branching and distributed throughout the warmer parts of India [1]. The herb possesses cylindrical fragile stems which bloom during the months of November-December [2].

Phytosterols, terpenes, tannins, glycosides, flavonoids, phenolic compounds, amino acids, fixed oils, and so forth are important phytochemicals isolated from this plant [3]. The flowers are reported to contain isosalipurposide, luteolin, glucoside, lutein, delphinidin-3,5-diglucoside, and pigments [4]. The juice prepared from the leaves is considered as cooling agent and used to cure smallpox in the infants. Bruised leaves are externally applied to relieve painful inflammations and swellings [5]. The paste prepared from fresh leaves mixed with castor oil is reported to cure tinea capitis, a scaly fungoid infection on scalp. Roots are used as febrifuge and vermifuge by the tribal population in India [6, 7].* R. pectinata* have been claimed to exhibit antipyretic, anti-inflammatory, diuretic, analgesic, antifungal, and antimicrobial activities [4, 8, 9].

Recently, due to high demand by the drug manufacturers, this plant has been overexploited. The natural population of* R. pectinata* is decreasing in the forests. Therefore, conservation activities have been initiated by the Wildlife Institute of India [10].

The transfer of vegetative phase to reproductive phase is a complicated and fascinating process in the life cycle of the plants. Environmental factors, photoperiod, and genetic makeup of the plant are responsible for flowering phenomena [11]. Reproductive phase can be induced by the artificial manipulation of these factors under* in vitro* controlled environment [12].* In vitro* flowering can be induced artificially by altering the concentration of plant growth regulators, different light qualities, photoperiods, and so forth [13–18]. The* in vitro* approach enables early flowering in the plants and could help in understanding the suitable sites of tissues for the flower induction.

The* in vitro* growing plants develop inefficient stomata, reduced epicuticular wax, and photosynthetic tissues; these features promote low regulation mechanism of water loss [19]. Therefore, the micropropagated plants failed to survive in the greenhouse and field trials [20]. Distribution and forms of trichomes, stomata, and venation pattern may vary depending on the environmental conditions [21]. The micromorphological studies of leaves could help in understanding the developmental changes in the leaf anatomy when the plants are cultured in the laboratory conditions.

Till date, the bioprospection reports on* R. pectinata* are restricted to the biological activities and isolation of different chemical compounds only. This is the first report on development of micropropagation protocol as conservation strategy and* in vitro* flowering in* R. pectinata* using nodal shoot segments.

## 2. Materials and Methods

### 2.1. Plant Material and Culture Initiation

Actively growing young shoots of* R. pectinata* were harvested from the forest areas of South India. The nodal segments were dissected with two nodes, rinsed in running tap water, and immersed in 2% Tween® 20 detergent solution for 15 min. The explants were then treated with 0.1% (w/v) Bavistin (systemic fungicide, BASF Pvt. Ltd., India) solution for 5 min and washed thoroughly in sterile distilled water. Finally the explants were surface-sterilized using 0.1% (w/v) solution of mercuric chloride for 4-5 min and rinsed with autoclaved distilled water for 5-6 times to remove the traces of sterilant. The sterilized explants were inoculated vertically on culture medium after trimming both the cut ends.

### 2.2. Culture Medium and Culture Conditions

Murashige and Skoog [22] (MS) medium consists of 30 g L^−1^ sucrose, 8.0 g L^−1^ agar, and additives (ascorbic acid 50 mg L^−1^, citric acid, adenine sulphate, and arginine each 25 mg L^−1^) which was used to establish the cultures* in vitro*. pH of the medium was adjusted to 5.8 using 0.1 N NaOH or HCl. Culture vessels with medium were capped using cotton plugs or polycarbonate caps and autoclaved at 121°C and 104 kPa for 15 min. The cultures were initiated using MS medium supplemented with 6-benzylaminopurine (BAP) and kinetin (Kin) ranging from 0.5 to 3.0 mg L^−1^. The cultures were incubated at 25 ± 2°C temperature for the photoperiod of 16 hr with 30 *μ*mol m^−2^ s^−1^ Spectral Flux Photon Density (SFPD) light provided by cool white fluorescent tubes.

### 2.3. *In Vitro* Multiplication of Shoots

The explants with fresh shoots from the initiation stage were cut into small nodal segments containing two nodes and subcultured on multiplication medium, augmented with various concentrations and combinations of BAP, Kin (0.0–2.0 mg L^−1^), and indole-3 acetic acid (IAA, 0.1–0.4 mg L^−1^). The subculture process was continuously repeated for up to four subcultures (each of four-week duration) to study the effect of subculture duration and growth hormones for stable multiplication of the shoots. The culture observations were recorded and the number and height of shoots were calculated after 4 weeks.

### 2.4. *In Vitro* Flowering

To induce flowering* in vitro*, the healthy shoots from third-fourth subculture stage of multiplication phase were isolated and inoculated to half strength MS medium containing growth hormones as described in multiplication stage and maintained under different photoperiods (12/12 hr/d, 16/8 hr/d, and 8/16 hr/d).

### 2.5. *In Vitro* Rooting of Shoots and Hardening of Plantlets

Healthy and mature elongated shoots measuring the size of 3.0–4.0 cm were harvested from the fourth subculture stage of multiplication. These shoots were dissected and transferred to different strengths of MS medium augmented with IAA, indole-3 butyric acid (IBA), and *α*-naphthalene acetic acid (NAA) individually to study the efficacy of auxins and medium strength in induction of roots* in vitro*. The entire rooting experiment was kept under diffused light area for a week and then shifted to the photon rich area of the culture room.

After four weeks of incubation, the well-rooted shoots were separated from the culture vessels and thoroughly washed with tap water. The plantlets with healthy roots were transferred to disposable paper cups (Vandana Paper Products, Chennai, India) containing 55 g sterile Soilrite®, moistened with one-fourth MS salts solution, and maintained in high humidity (80%) area of the greenhouse. After two months, the acclimatized plantlets were shifted to earthen pots containing autoclaved sand and garden soil (1 : 1). The plantlets were further maintained in shade house for two months and eventually shifted to the field.

### 2.6. Foliar Micromorphological Evaluation

*In vitro* regenerated leaves were used to study the vein density, venation pattern, and types of trichomes at rooting stage after 4 weeks of incubation. The entire leaves were preserved in formalin acetic acid (FAA) solution and cleared in 70% ethanol (v/v) to remove chlorophyll content, bleached with 5% (w/v) NaOH and rinsed in water until the plant material is free from NaOH. The leaves were then stained with 1% safranin (Loba Chemie, India) and mounted in water and examined under the microscope (Labomed iVu 3100, USA).

### 2.7. Data Collection and Statistical Analysis

Experiments were laid out in completely randomized block design [23] and repeated thrice. Each treatment consisted of minimum ten replicates. The observations on response of explants, number of shoots and their height, percentage of rooted shoots, and root length were recorded after a regular time interval of 4 weeks. Analysis of variance and Duncan's multiple range tests were used for comparison among resulting mean values.

## 3. Results and Discussion

### 3.1. Establishment of Cultures

Surface sterilization of the explants for 4 min and trimming both the cut ends reduced the rate of contamination. Bud breaking from nodal meristems was observed in all the explants with all the concentrations of growth regulators within 7 days of inoculation, whereas no shoots were developed on MS basal medium devoid of growth regulators.

**Figure 1:**
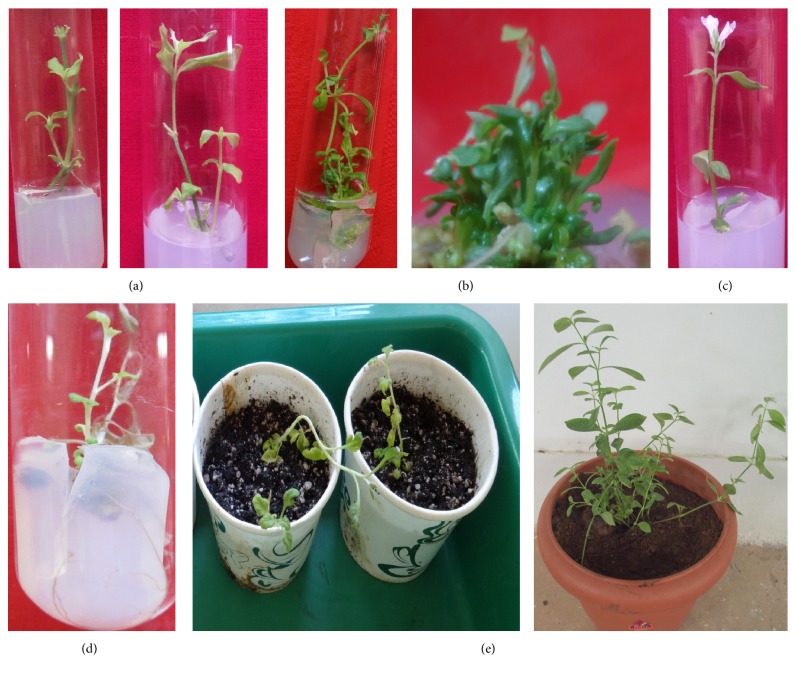
(a) Induction of shoots from the nodal meristems of the explants. (b) Multiplication of shoots of* R. pectinata*. (c)* In vitro* flowering in* R. pectinata*. (d) Induction of roots from the* in vitro* regenerated shoots in half strength MS medium with IBA. (e) Stages in hardening of plantlets in the greenhouse.

The percentage of bud breaking from nodal explants was increased while increasing the concentration of cytokinins, both BAP (up to 1.0 mg L^−1^) and Kin (up to 1.5 mg L^−1^), and decreased with higher concentrations. However, the percentage of culture responses was low on Kin (76%) compared to BAP (98%). Maximum 3.2 shoots with 4.3 cm average length resulted from MS medium augmented with BAP alone at 1.0 mg L^−1^ (Table 1). Benzylaminopurine was found better than Kin in terms of percentage response as well as number of shoots per explant. The superiority of BAP over Kin for bud break as well as shoot growth and multiplication has also been reported in* Cyclea peltata* [24] and* Morinda citrifolia* [25]. The number of shoots induced per explant varied in all the treatments. The length of axillary shoots regenerated from explants varied from 1.5 to 4.3 cm on MS medium with all the concentrations of BAP (Figure 1(a)). Only two shoots with 2.2 cm length were recorded with Kin, and the shoot lengths were varied from 1.7 to 2.8 cm. Increased concentrations of cytokinins inhibited the overall response of explants. This type of adverse effects of higher concentration of cytokinins at initiation stage has been reported in* Celastrus paniculatus* [26],* Caralluma edulis* [27], and* Passiflora foetida* [28].

### 3.2. Shoot Multiplication

The newly developed young shoots with explants were harvested and subcultured on fresh MS medium supplemented with BAP, Kin, and IAA. The response of explants at initiation stage indicated the significance of BAP in the multiplication medium. But significant increase in number of shoots was observed when the medium was incorporated with Kin and IAA along with BAP. The addition of IAA with BAP and Kin further improved the rate of shoots multiplication. Kataria and Shekhawat [29] reported the efficacy of BAP and IAA in shoot multiplication of* Rauvolfia serpentina*.

Maximum number of shoots (13.2) was observed on MS medium supplemented with 0.5 mg L^−1^ each of BAP and Kin and 0.1 mg L^−1^ IAA in this study (Table 2 and Figure 1(b)). The positive response and number of shoots with maximum length per culture showed a continuous increase in each subculture up to the fourth subculture, and thereafter it decreased. Repeated subcultures resulted in increase in number of multiple shoots. Recently Shekhawat et al. [25] and Patel et al. [27] had successfully used this technique to increase the shoot numbers in* M. citrifolia* and* Caralluma edulis*. They observed amplification of shoot numbers up to the third subculture stage. Similar observations were also made in different species such as* Pterocarpus santalinus* [30],* Justicia gendarussa* [31], and* Eulophia nuda* [32].

### 3.3. *In Vitro* Flowering in Cultures

Medium strength, growth regulators, and specific photoperiod had significant impact on* in vitro* flowering. The shoots (5-6 cm length) isolated from third subculture on half strength MS medium supplemented with 0.5 mg L^−1^ each of BAP and Kin + 0.1 mg L^−1^ IAA only responded in flowering. Among the photoperiods studied for flower induction* in vitro*, 12/12 hr light/dark photoperiod with 30 *μ*mol m^−2^ s^−1^ SFPD light intensity was found best. The* in vitro* induced flowers were morphologically similar to those of the plants in the field. Similar reports were published in* Vitex negundo* [33] and* Ipomoea sepiaria* [18]. The effect of photoperiod on* in vitro* flowering has also been studied in* Psygmorchis pusilla* [12]. About 80% shoots were responded in* in vitro* flowering in the present study (Table 3). The* in vitro* shoots end with the single inflorescence as in the naturally grown plants (Figure 1(c)).

### 3.4. *In Vitro* Rooting of Shoots and Hardening of Plantlets

The healthy shoots measuring 5-6 cm in length were used to induce roots by culturing on auxins supplemented medium. The shoots separated from third subculture responded better in this experiment. Maximum response in root induction was achieved when half strength MS medium was supplemented with 2.0 mg L^−1^ IBA. About 95% of shoots were rooted with 4.9 roots per shoot (3.5 cm length) on this medium combination (Table 4 and Figure 1(d)). IBA was found better in root induction response and number and length of roots compared to all concentrations of IAA and NAA evaluated for rooting efficiency. Maximum 2.8 roots with 2.14 cm length were induced with 67% response on MS medium incorporated with 1.0 mg L^−1^ NAA. But 3.6 roots with 2.0 cm length were recorded with 3.0 mg L^−1^ IAA. The superiority of IBA among other auxins has been reported in* M. citrifolia* [25] and* Ipomoea sepiaria* [18].

After four weeks of incubation in rooting medium, the* in vitro* rooted plantlets were separated from culture vessels and the remnants of medium were removed cautiously and placed in autoclaved Soilrite. These were initially moistened with one-fourth MS salts solution and maintained in high humidity area of the greenhouse for two months. The acclimatized plantlets were shifted to earthen pots (Figure 1(e)) containing autoclaved sand and garden soil (1 : 1). Finally the hardened plantlets were shifted to the field.

### 3.5. Foliar Micromorphological Study

**Figure 2:**
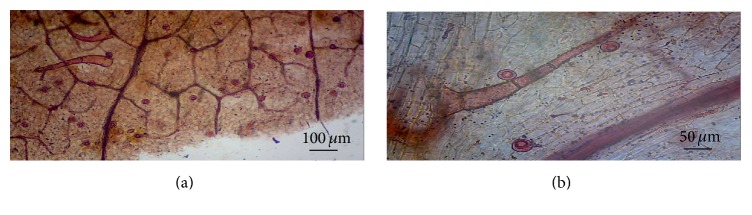
(a) Venation pattern in leaves of* in vitro* raised shoots; (b) multicellular, nonglandular trichome (*scale bar* 50.0 *µ*m, 100.0 *µ*m).

The epidermal cells of the leaves were large and thick and possessed characteristic diacytic stomata surrounded by two distinct subsidiary cells at the two poles of the guard cells. Trichomes were observed along with the veins and secondary veins. Two types of uniseriate trichomes, unicellular glandular and multicellular nonglandular trichomes, were observed. The glandular trichomes were spherical in shape and consisted of unicellular head and subsessile stalk. The nonglandular trichomes were long and consisted of thick-walled broader cells with cuneate base and pointed apex. Glandular trichomes were more frequent than the nonglandular trichomes (Figures 2(a) and 2(b)). Amit and Daniel [34] reported the presence of diacytic stomata and glandular and nonglandular trichomes in* Rungia repens.* The presence of trichomes was considered as indicative on the concentration of secondary metabolites and as important biomarkers in plant identification [35]. Glandular trichomes with empty cells were also reported in* Rungia repens* by Dipa and Daniel [36].

The cystoliths present in the petioles of* R. pectinata* were reported by A. M. Patil and D. A. Patil [37] and Metcalfe and Chalk [38] and their significance was studied by Ahmad [39] but no cystoliths were observed in the present experiment. Large and distinct vein-islets were reported with polygonal areoles (islets). The* in vitro* environment at rooting stage enhances the area of polygonal regions by meristematic growth and each becomes subdivided into loops. The vein-islets adjacent to margin do not show loop formation (Figure 2(a)). It is understood that the acclimatization processes further enhance the vein-islet density and favor the plantlet growth in the field. The architectural properties of leaf venation are related to functional aspects. The leaf venation pattern of* R. pectinata* indicates strong selective pressures acting on the architectural arrangement of the conducting bundles of the leaf at rooting stage. Pospíšilová [40] and Wang et al. [41] reported that the application of cytokinins in culture medium significantly affects rate of transpiration.

Wax deposition, transpiration from leaves, stomatal functionality, and changes in structure after transplantation in the* ex vitro* and field conditions were studied in number of plants [42–44]. So the knowledge of various functional features of leaf venation pattern could enhance the survival rate of the micropropagated plants in the field.

### 3.6. Conclusion

Tissue culture protocol has been successfully developed for* R. pectinata* in this study. The regenerated shoots were subjected to induction of* in vitro* flowering. The plantlets were hardened and field-transferred. The foliar micromorphological study was conducted to understand the developmental changes during* in vitro* culture at rooting stage.

## Figures and Tables

**Table 1:** Effect of cytokinins on induction of shoots from nodal segments of *R. pectinata*.

Conc. of cytokinins (mg L^−1^)		Response (%)	Shoot number (mean ± SD)	Shoot length (cm) (mean ± SD)
BAP	Kin			
0.0	0.0	0	0.0 ± 0.00^a^	0.0 ± 0.00^a^
0.5	—	75	1.2 ± 0.11^b^	3.0 ± 0.20^e^
1.0	—	98	3.2 ± 0.10^f^	4.3 ± 0.13^d^
1.5	—	86	1.7 ± 0.21^d^	3.2 ± 0.21^d^
2.0	—	72	1.0 ± 0.24^b^	2.8 ± 0.14^b^
3.0	—	60	1.2 ± 0.17^b^	1.5 ± 0.21^b^
—	0.5	59	1.5 ± 0.20^c^	1.8 ± 0.10^c^
—	1.0	74	2.0 ± 0.15^e^	2.2 ± 0.26^d^
—	1.5	76	1.2 ± 0.24^b^	2.8 ± 0.14^c^
—	2.0	65	1.4 ± 0.40^c^	2.0 ± 0.31^b^
—	3.0	52	1.0 ± 0.16^b^	1.7 ± 0.19^b^

*Note.* Mean separation was analyzed by ANOVA using SPSS software (ver. 16.0); the values represented in corresponding column followed by same letters are not significantly different according to DMRT at *P* < 0.05.

**Table 2:** Effect of different concentrations of cytokinins (BAP and Kin) combined with IAA on multiplication of shoots.

Conc. of BAP and Kin (mg L^−1^)	Conc. of IAA (mg L^−1^)	Shoot number (mean ± SD)	Shoot length (cm) (mean ± SD)
0	0	0.0 ± 0.0^a^	0.0 ± 0.0^a^
0.25	—	6.3 ± 1.2^c^	5.3 ± 0.4^g^
0.5	—	9.5 ± 1.9^d^	5.9 ± 0.3^d^
1.0	—	7.3 ± 1.0^c^	4.2 ± 0.6^e^
1.5	—	6.6 ± 1.3^b^	4.7 ± 0.1^b^
2.0	—	5.4 ± 1.0^h^	5.0 ± 0.5^h^
0.5	0.1	13.2 ± 1.6^f^	5.2 ± 0.0^f^
0.5	0.2	9.3 ± 0.9^c^	4.5 ± 0.7^c^
0.5	0.3	8.1 ± 1.0^b^	4.0 ± 0.2^b^
0.5	0.4	5.2 ± 0.7^h^	3.5 ± 0.3^b^

*Note.* Mean separation was analyzed by ANOVA using SPSS software (ver. 16.0); the values represented in corresponding column followed by same letters are not significantly different according to DMRT at *P* < 0.05.

**Table 3:** Effect of photoperiod on *in vitro* flowering of *R. pectinata* on half strength MS medium after 4 weeks of culture.

Photoperiod (hr/d) (light/dark)	Percentage of shoots producing inflorescence
12/12	80
16/8	68
8/16	00

**Table 4:** Effect of auxins on *in vitro* root induction of *R. pectinata* on half strength MS medium.

PGR and its concentration (mg L^−1^)	Response (%)	Root number (mean ± SD)	Root length (cm) (mean ± SD)
0	0	0.00 ± 0.00^a^	0.00 ± 0.00^a^

IAA			
1.0	60	2.5 ± 0.16^a^	1.10 ± 0.24^b^
2.0	72	2.8 ± 0.21^c^	2.80 ± 0.17^e^
3.0	69	3.6 ± 0.29^e^	2.00 ± 0.22^c^
4.0	60	2.4 ± 0.10^b^	1.28 ± 0.38^b^

IBA			
1.0	91	3.7 ± 0.13^e^	2.71 ± 0.29^e^
2.0	95	4.9 ± 0.22^f^	3.50 ± 0.15^g^
3.0	87	3.2 ± 0.10^d^	3.00 ± 0.40^f^
4.0	70	2.6 ± 0.07^c^	2.50 ± 0.23^d^

NAA			
1.0	67	2.8 ± 0.21^c^	2.14 ± 0.41^c^
2.0	75	2.6 ± 0.30^c^	2.37 ± 0.23^e^
3.0	68	2.7 ± 0.33^c^	2.15 ± 0.45^c^
4.0	61	2.0 ± 0.24^b^	1.40 ± 0.19^b^

*Note.* Mean separation was analyzed by ANOVA using SPSS software (ver. 16.0); the values represented in corresponding column followed by same letters are not significantly different according to DMRT at *P* < 0.05.

## Abbreviations

BAP: 6-Benzylaminopurine
Kin: Kinetin
IAA: Indole-3-acetic acid
IBA: Indole-3-butyric acid
NAA: *α*-Naphthalene acetic acid
MS medium: Murashige and Skoog [22] medium
SFPD: Spectral Flux Photon Density
FAA: Formalin acetic acid.
